# Supplementary material for: A late B lymphocyte action in dysfunctional tissue repair following kidney injury and transplantation
Source: Nat Commun. 2019 Mar 11;10:1157. doi: 10.1038/s41467-019-09092-2 (PMC6411919; doi:10.1038/s41467-019-09092-2)
Supplement: Supplementary file 1 — Supplementary Information [file 41467_2019_9092_MOESM1_ESM.pdf]

Supplementary Information for

"A late B lymphocyte action in dysfunctional tissue repair following kidney injury and transplantation"

(Cippà et al., 2019)

Supplementary figure 1

a b

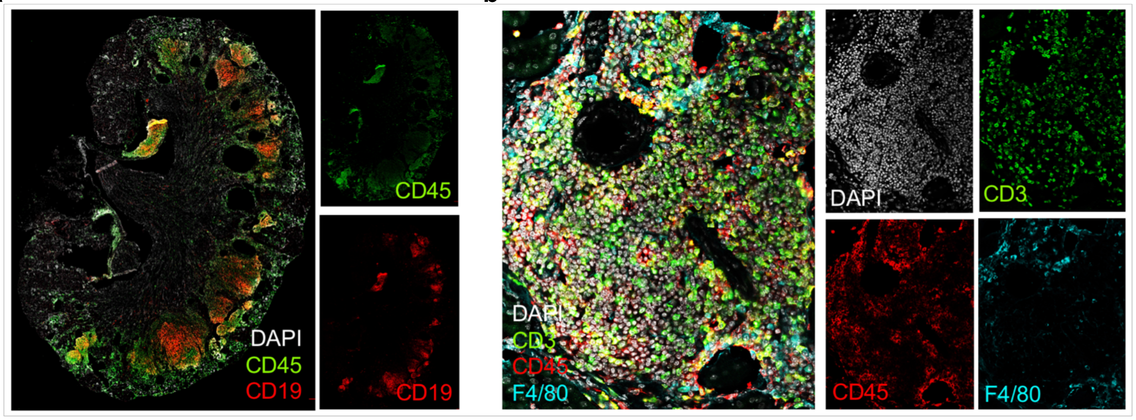

c

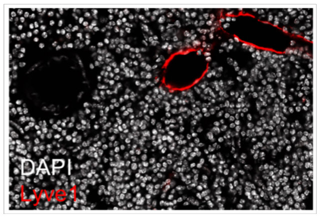

Suppl. figure 1 Characterization of renal ectopic lymphoid structures. Immunostaining of consecutive sections obtained from a representative mouse kidney 6 months after IRI (n=3-4/group). CD19: B cells; CD45: immune cells; CD3: T cells; F4/80: macrophages. CD45R: B cells (and a subset of T cells, s. suppl. figure 2); Lyve1: lymphatic vessels.

Supplementary figure 2

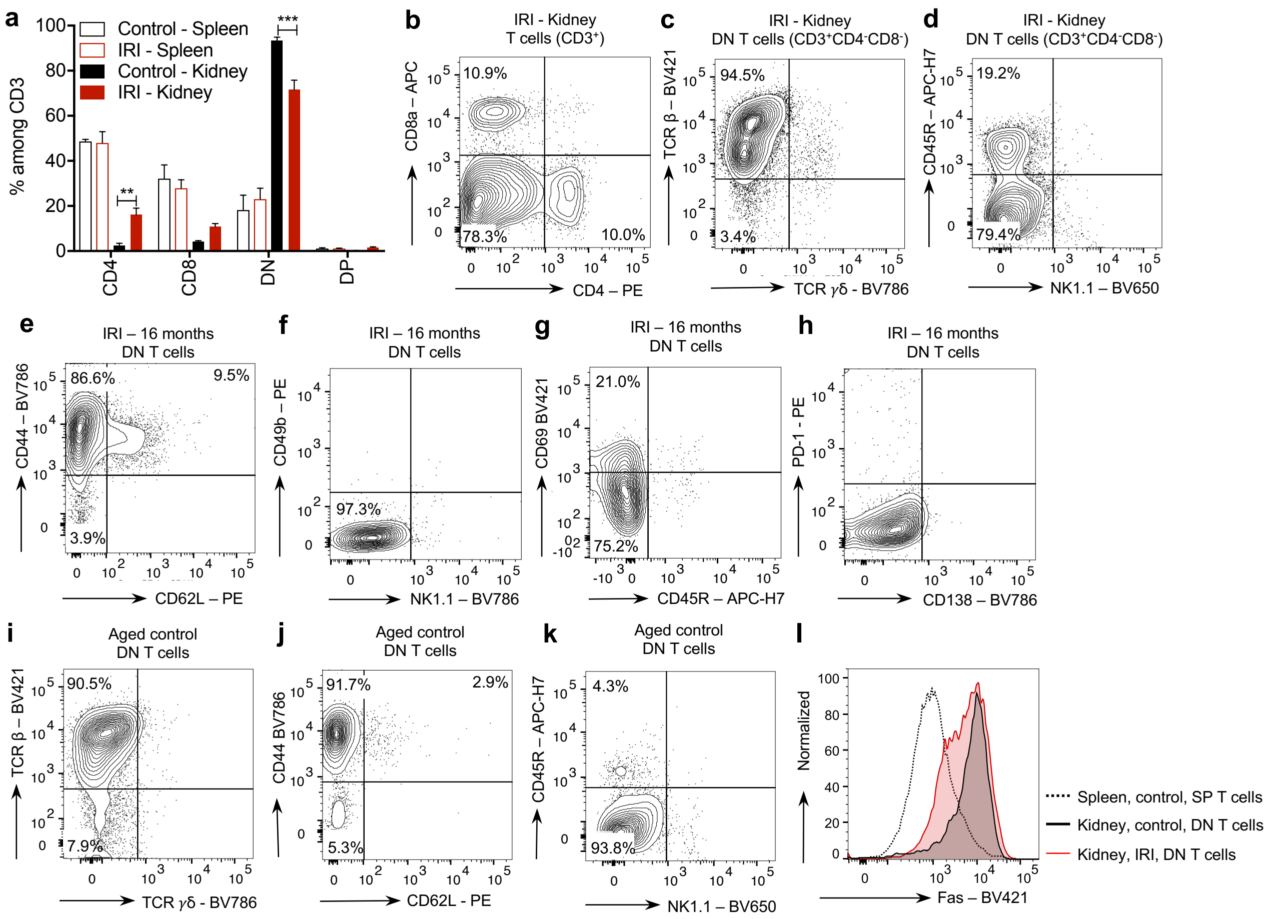

Suppl. figure 2 Characterization of renal T cells after IRI. Flow cytometric analysis of leukocytes isolated from the kidney 16-18 months after IRI or from aged control mice, as indicated (representative examples are shown, n=6 in IRI and n=3 in controls). (a-b) General characterization of $CD3^+$ T cells according to the expression of CD4 and CD8. DN: double negative, DP: double positive; Mann-Whitney test, ** $P<0.01$, *** $P<0.001$. (c-h) Further characterization of DN T cells. TCR: T cell receptor; NK1.1 and CD49b: natural killer cell markers; CD45R is the isoform of CD45 typically expressed by B cells, and variably detectable on DN T cells. (i-k) Analogous characterization of renal DN T cells in age-matched control mice, showing a similar phenotype as detected after IRI, but not CD45R expression. (l) Representative flow cytometry analysis of Fas expression on T cells: DN T cells isolated from the kidney of both IRI and control mice, but not single positive (CD4 or CD8 positive) T cells from the spleen expressed high levels of the apoptosis receptor Fas. General gating strategy is presented in Suppl. Fig. 5.

Supplementary figure 3

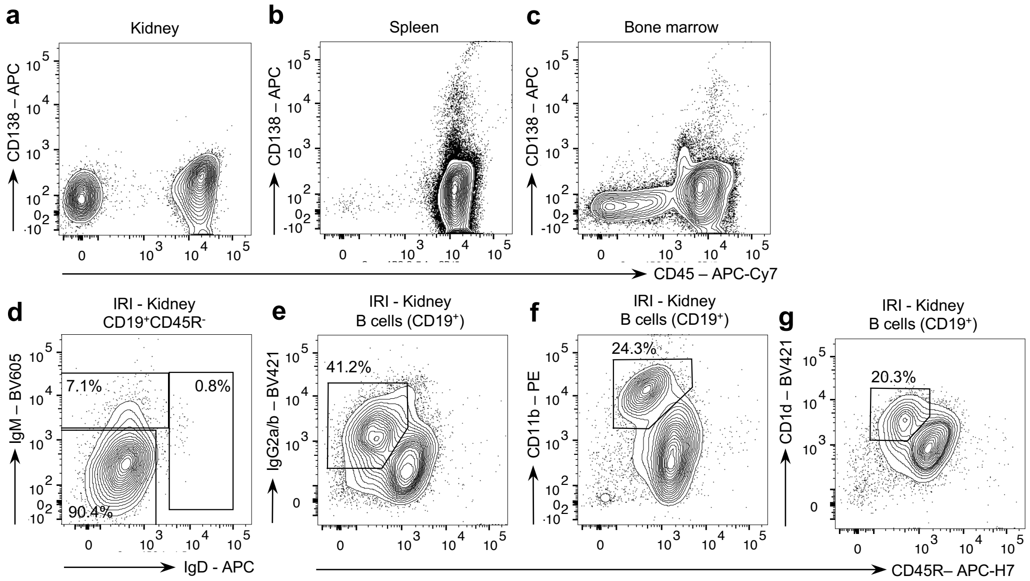

Suppl. figure 3 Characterization of renal B lymphocytes after IRI. Representative examples of flow cytometric analysis on leukocytes isolated from the kidney, the spleen and the bone marrow 16-18 months after IRI. (a-c) Controls to exclude that the absence of CD138 detection in renal B cells is related to the sorting procedure (compare with Fig. 4). Unsorted leukocytes isolated from the kidney, the spleen and the bone marrow and were directly stained for CD45 and CD138 without any additional manipulation. CD138 is detectable in the spleen and in the BM but not in the kidney. (d-g) Characterization of CD45^{+}CD19^{+} B cells isolated from the kidney. (d) Only CD45R$^{-\text{ or dim}}$ cells are shown. (e-g) CD45R$^{-\text{ or dim}}$ cells are compared to CD45R^{+} cells. CD11b was reported in subsets of CD45R^{-} memory B cells (Driver et al., *J Immunol* 2001); and B cell expression of CD1d is involved in the pathogenesis of autoimmunity (Chaudhry et al., *J Immunol* 2014). General gating strategy is presented in Suppl. Fig. 5.

Supplementary figure 4

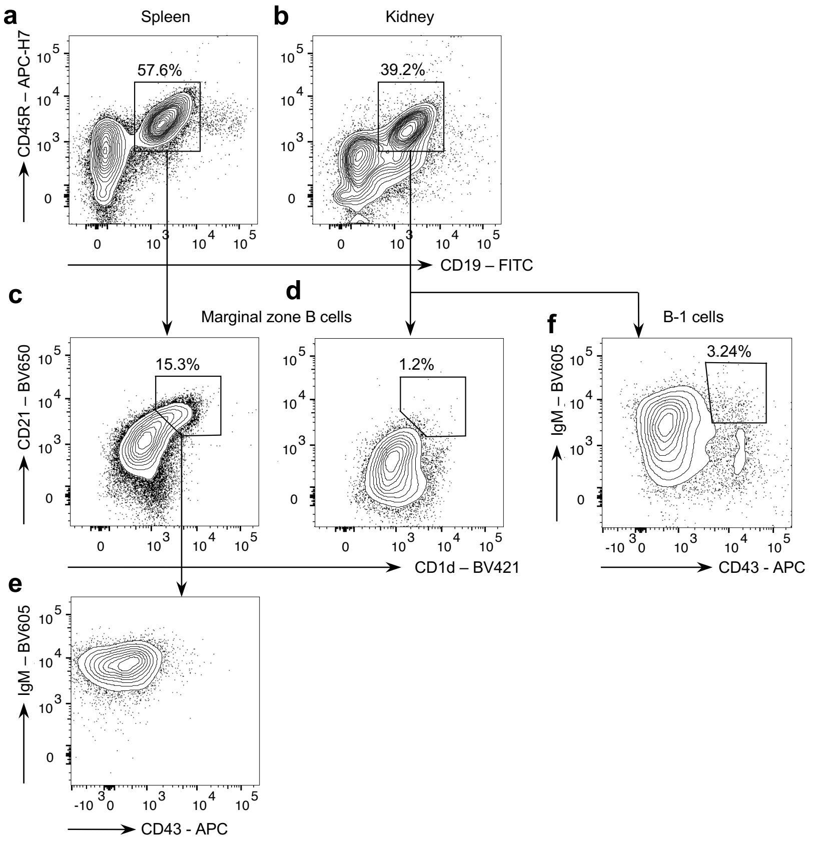

Suppl. figure 4 Flow cytometric analysis of leukocytes isolated from the kidney to investigate the presence marginal zone or B-1 cells after IRI. Cells isolated from the kidney (b,d,f) are compared with splenocytes (a,c,e). (c-e) Among $CD45^{+}CD19^{+}CD45R^{+}$ isolated from the kidney we did not detect $CD1d^{+}CD21^{+}$ marginal zone B cells. In the spleen this B cell population was present and displayed the additional markers IgM and CD43. (f) $CD43^{+}IgM^{+}$ B-1 cells were not present in the kidney. General gating strategy is presented in Suppl. Fig. 5.

Supplementary figure 5

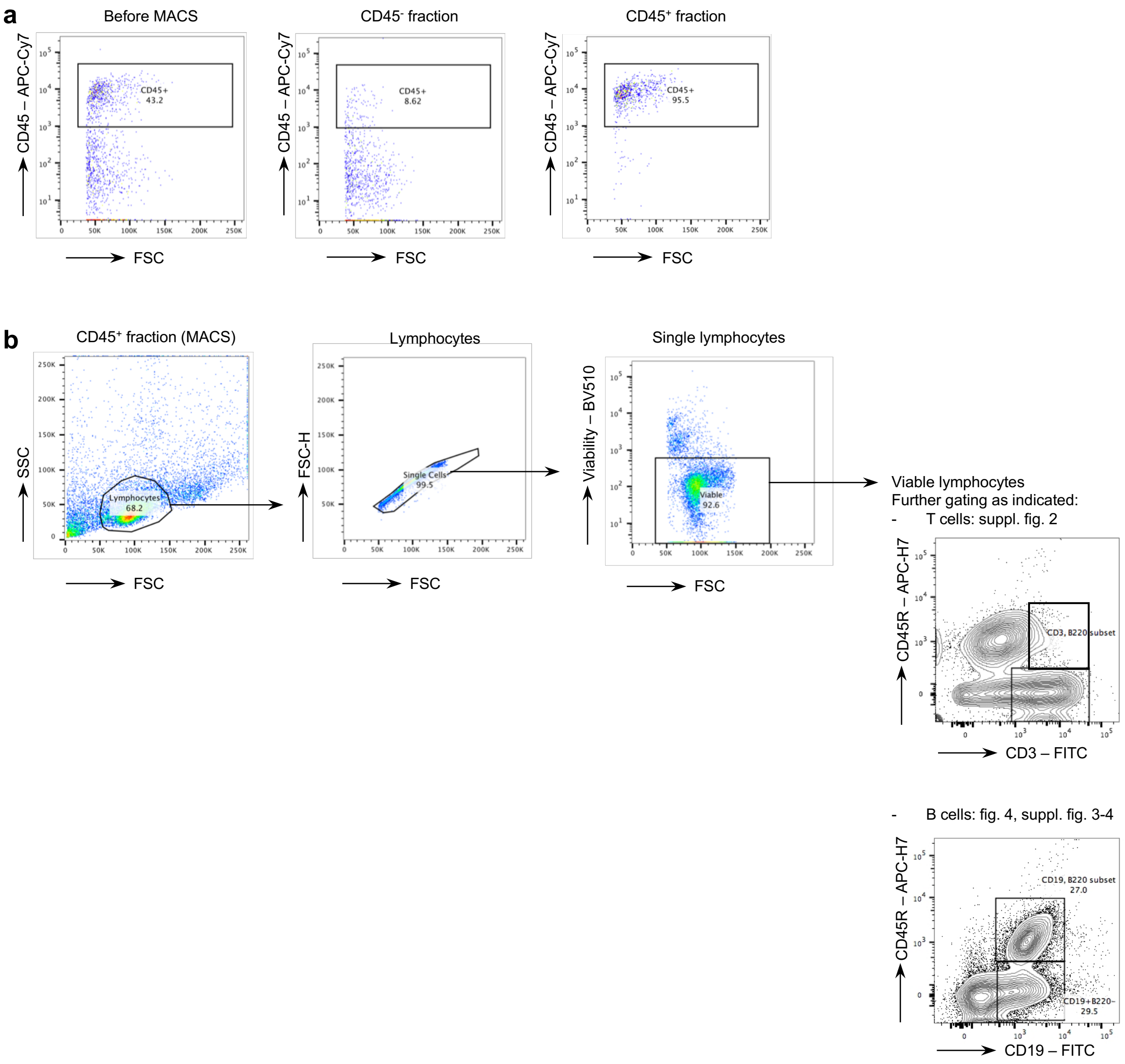

Suppl. figure 5 Sorting control and general gating strategy for FACS analysis. (a) Representative example of cell separation obtained by magnetic cell sorting (MACS) with CD45 beads on cells isolated from the renal tissue. In the CD45⁺ fraction, >95% of the cells were confirmed by FACS to display CD45. (b) Representative example of the general sequential gating strategy applied to cells isolated from the kidney, the spleen and the bone marrow. Depending on the gate indicated in the main figures, further analyses were performed on all CD3⁺, all CD19⁺ or CD19⁺CD45R⁻ cells.

Supplementary Table 1 Baseline clinical characteristics in CKI and non-CKI group

LD: living donor, DBD: donation after brain death, DCD: donation after cardiac death. Banff criteria: ah: arteriolar hyalinosis, cg: chronic glomerular lesions, ci: chronic interstitial lesions, ct: chronic tubular lesions, cv: chronic vascular lesions. ATN: acute tubular necrosis. TAC: tacrolimus, MMF: mycophenolate mofetil, CS: corticosteroids.

	CKI-group (N=15)	Other patients (N=24)	P-value
**Baseline**			
Donor type (LD / DBD / DCD)	1 / 8 / 6	3 / 15 / 6	0.57
Donor age (years)	47.7 ± 16.2	47.5 ± 15.3	0.85
Donor sex (male / female)	10 / 5	8 / 16	0.04*
Donor creatinine (mg/dl)	0.73 ± 0.28	0.77 ± 0.23	0.70
Recipient age	49.8 ± 15.5	53.5 ± 12.1	0.53
Recipient sex	7 / 8	17 / 7	0.13
Recipient end stage renal disease diagnosis			
- Diabetes	0	1	
- Hypertension	1	4	
- Glomerulonephritis / Interstitial nephritis	5	8	
- Congenital / Polycystic kidney disease	5	3	
- Other	0	4	
- Undefined	4	4	
Previous renal transplant	0	3	
Cold ischemia time (h)	12.7 ± 6.1	11.7 ± 5.2	0.32
Delayed graft function (N)	3	1	0.11
HLA-mismatches (mean)	2.8 / 6	3 / 6	0.71
Pre-transplant HLA sensitization	1	2	0.84
Histology (according to Banff)			
- ah (0 / 1 / >1)	13 / 2 / 0	16 / 6 / 2	
- cg (0 / 1 / >1)	15 / 0 / 0	24 / 0 / 0	
- ci (0 / 1 / >1)	11/ 3 / 1	15 / 8 / 1	
- ct (0 / 1 / >1)	6 / 9 / 0	11 / 13 / 0	
- cv (0 / 1 / >1)	12 / 2 / 1	22 / 2 / 0	
- mm (0 / 1 / >1)	15 / 0 / 0	24 / 0 / 0	
- ATN score (2 or higher)	6	9	
- % sclerotic glomeruli	7.3	7.9	
Induction therapy<br>- Anti-CD25 therapy	6	9	
Maintenance immunosuppression<br>- TAC + MMF + CS (%)	15	24	

Supplementary Table 2 Patients with clinical evidence of B cell mediated allograft damage or donor-specific sensitization.

**Patient ID**	**Group**	**Evidence**
#01	CKI	New DSA 2 years after transplant
#05	CKI	New DSA 1 year after transplant
#22	CKI	Peritubular capilaritis (ptc++) and glomerulitis (g++) in indication biopsies (day 51 and day 493 after transplant)
#13	*CKI*	*Sensitized patients, acute antibody mediated rejection in the first month after transplant.* *Note: in this case the B cell response is not a consequence of a dysfunctional kidney repair and is not considered as an example of late B cell immunity.*

DSA: donor specific antibody
