# Supplementary material for: A late B lymphocyte action in dysfunctional tissue repair following kidney injury and transplantation
Source: Nat Commun. 2019 Mar 11;10:1157. doi: 10.1038/s41467-019-09092-2 (PMC6411919; doi:10.1038/s41467-019-09092-2)
Supplement: Supplementary file 3 — Description of Additional Supplementary Files [file 41467_2019_9092_MOESM3_ESM.pdf]

Description of Additional Supplementary Files

Supplementary Data 1

List of the genes differentially expressed in the CKI group in comparison to the non-CKI group in protocol biopsies obtained 12 months after transplantation.

Supplementary Data 2

Multiple t-test on 186 genes previously validated to detect allograft rejection comparing the CKI and non-CKI groups in protocol biopsies obtained 3 months after transplantation.

Supplementary Data 3

Multiple t-test on 29 genes previously validated to detect acute kidney injury rejection comparing the CKI and non-CKI groups in protocol biopsies obtained 3 months after transplantation.
