# Supplementary material for: A late B lymphocyte action in dysfunctional tissue repair following kidney injury and transplantation
Source: Nat Commun. 2019 Mar 11;10:1157. doi: 10.1038/s41467-019-09092-2 (PMC6411919; doi:10.1038/s41467-019-09092-2)
Supplement: Supplementary file 7 — Reporting Summary [file 41467_2019_9092_MOESM7_ESM.pdf]

nature research

Corresponding author(s): *Double-blind peer review submissions: write DBPR and your manuscript number here instead of author names.*

Reporting Summary

Nature Research wishes to improve the reproducibility of the work that we publish. This form provides structure for consistency and transparency in reporting. For further information on Nature Research policies, see Authors & Referees and the Editorial Policy Checklist.

Statistical parameters

When statistical analyses are reported, confirm that the following items are present in the relevant location (e.g. figure legend, table legend, main text, or Methods section).

n/a	Confirmed	
☐	☐	The exact sample size (n) for each experimental group/condition, given as a discrete number and unit of measurement
☐	☒	An indication of whether measurements were taken from distinct samples or whether the same sample was measured repeatedly
☐	☒	The statistical test(s) used AND whether they are one- or two-sided<br>*Only common tests should be described solely by name; describe more complex techniques in the Methods section.*
☒	☐	A description of all covariates tested
☐	☒	A description of any assumptions or corrections, such as tests of normality and adjustment for multiple comparisons
☐	☒	A full description of the statistics including central tendency (e.g. means) or other basic estimates (e.g. regression coefficient) AND variation (e.g. standard deviation) or associated estimates of uncertainty (e.g. confidence intervals)
☐	☒	For null hypothesis testing, the test statistic (e.g. F, t, r) with confidence intervals, effect sizes, degrees of freedom and P value noted<br>*Give P values as exact values whenever suitable.*
☒	☐	For Bayesian analysis, information on the choice of priors and Markov chain Monte Carlo settings
☒	☐	For hierarchical and complex designs, identification of the appropriate level for tests and full reporting of outcomes
☒	☐	Estimates of effect sizes (e.g. Cohen's d, Pearson's r), indicating how they were calculated
☐	☒	Clearly defined error bars<br>*State explicitly what error bars represent (e.g. SD, SE, CI)*

Our web collection on statistics for biologists may be useful.

Software and code

Policy information about availability of computer code

Data collection	The RNAseq reads were aligned to the Ensembl top-level assembly with STAR version 2.0.4b. Gene counts were derived from the number of uniquely aligned unambiguous reads by Subread:featureCount version 1.4.5. Transcript counts were produced by Sailfish version 0.6.3. Sequencing performance was assessed for total number of aligned reads, total number of uniquely aligned reads, genes and transcripts detected, ribosomal fraction known junction saturation and read distribution over known gene models with RSeQC version 2.3.
Data analysis	Gene correlation analysis and feature correlation heatmaps were performed on the PIVOT platform, developed by the Kim Lab (http://kim.bio.upenn.edu/software/pivot.shtml). Dimension reduction analysis was performed with t-distributed stochastic neighbor embedding (t-SNE) by Rtsne package in R. Sample correlation analyses were performed on PIVOT10 or on Cluster (version 3.0) and visualized in TreeView (http://jtreeview.sourceforge.net). Box plots, scatter plots and histograms were generated with Prism 7.

For manuscripts utilizing custom algorithms or software that are central to the research but not yet described in published literature, software must be made available to editors/reviewers upon request. We strongly encourage code deposition in a community repository (e.g. GitHub). See the Nature Research guidelines for submitting code & software for further information.

Data

Policy information about availability of data

All manuscripts must include a data availability statement. This statement should provide the following information, where applicable:

- Accession codes, unique identifiers, or web links for publicly available datasets
- A list of figures that have associated raw data
- A description of any restrictions on data availability

Data are deposited on...

Field-specific reporting

Please select the best fit for your research. If you are not sure, read the appropriate sections before making your selection.

☒ Life sciences ☐ Behavioural & social sciences ☐ Ecological, evolutionary & environmental sciences

For a reference copy of the document with all sections, see nature.com/authors/policies/ReportingSummary-flat.pdf

Life sciences study design

All studies must disclose on these points even when the disclosure is negative.

Sample size	For studies in humans the required samples size was estimated based on previous experience and considering sample availability and costs. For studies in mice, sample size was calculated in consideration of pilot experiments.
Data exclusions	Human samples with insufficient RNA quality for RNAseq were excluded before sequencing.
Replication	Studies in humans were performed in one cohort and provided clinical data for hypothesis generation, to be verified in the mouse model. Mouse experiments were performed in 3 independent replicates for each time point and compared with sham operated mice (also in triplicates).
Randomization	Randomization is not applicable in the cohort study. Mice were randomly selected to be used for ischemia/reperfusion or sham operation.
Blinding	All computational analyses on human data were blinded. The team involved in the computational analysis was not informed about any clinical information until the end of the study, when computational and clinical data were matched. The histological evaluation was independent of the computational analysis and the pathologist was not informed about the results of the transcriptional analysis. Blinding was not possible in mouse experiments.

Reporting for specific materials, systems and methods

Materials & experimental systems

n/a	Involved in the study	
☒	☐	Unique biological materials
☐	☒	Antibodies
☒	☐	Eukaryotic cell lines
☒	☐	Palaeontology
☐	☒	Animals and other organisms
☐	☒	Human research participants

Methods

n/a	Involved in the study	
☒	☐	ChIP-seq
☐	☒	Flow cytometry
☒	☐	MRI-based neuroimaging

Antibodies

Antibodies used	Following antibodies were used in this study to recognize CD3 (rabbit, Abcam, ab16669), CD19 (rat, Invitrogen, 13-0194-82), CD21 (rabbit, Abcam, ab75985), CD31 (rat, BD, 553370), CD45 (goat, BD, 550280), CD45R (rat, BD, 557390), Cxcl13 (goat, R&D, AF470), F4/80 (rat, eBioscience, 14-4801), Havcr1 (goat, R&D, AF1817), Ltl lectin-FITC conjugate (Vector Laboratories, FL-1321), Lyve1 (goat, R&D, AF2125), Ki67 (rabbit, Novocastra, Ki67p-CE).
Validation	All antibodies were extensively validated in the McMahon lab before being used for this study.

Animals and other organisms

Policy information about studies involving animals; ARRIVE guidelines recommended for reporting animal research

Laboratory animals	We used C57BL/6CN male mice, purchased from Charles Rive, 10- to 12-week-old, 25- 28g,
Wild animals	The study did not involve wild animals.
Field-collected samples	The study did not involve field-collected samples.

Human research participants

Policy information about studies involving human research participants

Population characteristics	The characteristics of the study population are presented in Suppl Table 2.
Recruitment	The patients were randomly selected among patients with a full set of 4 available biopsies from a database at the University of Leuven. The selection process did not generate any relevant selection bias.

Flow Cytometry

Plots

Confirm that:

☒ The axis labels state the marker and fluorochrome used (e.g. CD4-FITC).

☒ The axis scales are clearly visible. Include numbers along axes only for bottom left plot of group (a 'group' is an analysis of identical markers).

☒ All plots are contour plots with outliers or pseudocolor plots.

☒ A numerical value for number of cells or percentage (with statistics) is provided.

Methodology

Sample preparation	The mouse was perfused with PBS until the kidney was visually blood-free. The kidney was removed, mechanically dissociated and then incubated at 37°C in digestion buffer (RPMI medium, 1 mg/ml type I collagenase, 10 g/ml DNAse, 10% FCS, 25 mM HEPES, penicillin and streptomycin). After 60 minutes the tissue was filtered through a 100 m nylon mash to remove remaining large fragments. CD45+ cells were positively sorted by magnetic cell separation in an autoMACS with anti-mouse CD45 beads (Miltenyi biotec, Cat# 130-053-301).
Instrument	Aria II, BD bioscience
Software	FlowJo 2, Version 1.1.0
Cell population abundance	Flow cytometry was used for analysis only.
Gating strategy	The gating strategy is indicated on each plot. In case of complex gating strategy, this is shown (Suppl fig. 4).

☒ Tick this box to confirm that a figure exemplifying the gating strategy is provided in the Supplementary Information.
